# Low metformin dose and its therapeutic serum concentration in prediabetes

**DOI:** 10.1038/s41598-021-91174-7

**Published:** 2021-06-03

**Authors:** Edyta Sutkowska, Paulina Fortuna, Jerzy Wisniewski, Karolina Sutkowska, Pawel Hodurek, Andrzej Gamian, Bernadetta Kaluza

**Affiliations:** 1 Department and Division of Medical Rehabilitation, Wroclaw Medical University, Borowska 213, 50-556 Wrocław, Poland; 2 Department of Medical Biochemistry, Wroclaw Medical University, Wrocław, Poland; 3 Faculty of Medicine, Wroclaw Medical University, Wrocław, Poland; 4 Hirszfeld Institute of Immunology and Experimental Therapy, Polish Academy of Sciences, Wroclaw, Poland; 5 Department of Internal Medicine, Endocrinology and Diabetology, Central Clinical Hospital of the Ministry of the Interior, Warsaw, Poland

**Keywords:** Pre-diabetes, Monosaccharides, Risk factors

## Abstract

This prospective study aimed to analyze whether the patients with pre-diabetes (pre-DM) reach the TC (therapeutic concentration) of the metformin during repeated, low, constant drug dose. The guidelines do not recommend any metformin dose for this group of patients. Based on the previous study after a dose of 1700 mg/day the patients seem to reach the therapeutic drug concentration, which guarantees the glycemic effect. Twenty patients with new-diagnosed pre-DM were treated with a 1500 mg/day regimen of the metformin for 15 weeks. The serum concentration of the drug was assessed by liquid chromatography-mass spectrometry technique at 6 and 15 week of the treatment. The correlation of the serum metformin concentration with BMI (body mass index) and patients’ weight was also performed. The mean metformin concentration was: 4.65 μmol/L (± 2.41) and 5.41 μmol/L (± 3.44) (p = 0.27) after 6 and 15 weeks of the treatment respectively. There was a positive correlation between the serum concentration of the metformin and body weight (but not BMI) in the 15th week of the therapy (p = 0.04)- the higher body weight the higher concentration of the metformin. Patients with pre-diabetes can be successfully treated with a low dose of metformin, to reach the drug’s therapeutic concentration. Body weight can impact the metformin serum concentration during long-term treatment what should be taken into consideration when choosing the dose because of its pleiotropic effect e.g. on the cardiovascular system via reduction of the oxidative stress and would be not connected with the drug’s hypoglycemic effect.

**ClinicalTrials.gov number**: NCT03398356; date of first registration: 01/07/2018.

## Introduction

The DPP (Diabetes Prevention Program)^1^ study showed that people who are at high risk for T2DM (type 2 diabetes mellitus) can prevent or delay the disease by lifestyle modification (diet and increased physical activity) or taking metformin by 58 and 31 percent, respectively, compared with placebo.

The DPPOS (Diabetes Prevention Program Outcomes Study)^2^, the follow-up program for DPP participants, confirmed that these individuals who were treated with metformin delayed in the development of T2DM by 18 percent compared with the “placebo group”, after 10 and 15 years of the observation. This was especially beneficial for those with higher baseline FG (fasting glucose) or HbA1c and women with a history of gestational diabetes mellitus^3^. The cost-effectiveness of the therapy was also noticed^4^. In the DPP, metformin also improved lipoprotein subfractions^5^, C-reactive protein and tissue plasminogen activator^6^ results. The metformin therapy also affected the incidence of metabolic syndrome (decreased by 17%) generally^7^ and the presence and severity of coronary artery calcium in men, compared to placebo^8^.

Based on this information as well as other small-scale studies, in 2016 the National Institute of Diabetes & Digestive & Kidney Diseases together with the National Heart Lung and Blood Institute and the National Cancer Institute, began funding a third phase of DPPOS. These part of the study is planned for 10 years, and researchers want to find out if people who are at high risk for type 2 diabetes and take metformin are at a lower rates of cardiovascular diseases and cancer because of its impact on the reduction of the e.g. oxidative stress.

The range of doses of the medicine that produces defined therapeutic effect without significant side effects is called the therapeutic window and means the amount of medicine that should result in the expected benefits (effectiveness of the drug). While in the case of expected simple effects such as lowering blood glucose, determining the therapeutic dose of the hypoglycemic agent seems relatively simple due to the easy assessment of effectiveness, it is more difficult to answer the question what we consider as the therapeutic dose for a drug with pleiotropic effect e.g. via antioxidant activity. The example is metformin because we expect not only the hypoglycemic effect but also the pleiotropic ones. This effect could be find not only within the group of patients with diabetes but also with pre-diabetes condition or maybe even in healthy population as is not related to glycemia.

Metformin has been demonstrated to reduce ischemic heart events compared to diet^9^, sulfonylurea treatments^10^ and versus placebo in insulin treated patients with T2DM^11^. The mentioned above DPP study was the first to demonstrate that metformin may have a beneficial effect on coronary atherosclerosis in a prediabetic population.

The average elimination half-life in plasma for regular form of the metformin is approximately 5 h. According to this information, to reach steady-state, dosing 2–3 times per day is recommended^12^. At usual clinical doses and schedules, metformin steady-state plasma concentrations are generally < 1.5 μg/ml, and the maximum drug plasma levels during controlled clinical trials do not generally exceed 5 μg/ml^13^.

Prescribing the lowest, target dosage of the metformin (1500 mg/day) to the patients with pre-DM we strongly believe that the drug reach the TC and thus e.g. protect them from diabetes. Previously, to check the metformin concentration, blood samples only from healthy people or patients with type 2 diabetes were analyzed. The therapeutic concentration for metformin is not well defined^14^ but could be find as range from 0.1 to 20.7 (median 4.5) μmol/L2 ^13,15–17^. To find if the smallest dose gives any pleiotropic benefit to the patients with pre-diabetes, long-term studies are needed. The first step to starting this story could be the assessment of the metformin therapeutic concentration in pre-diabetes population to whom the proper dose has not yet been established. The aim of our study is to assess do the patients with pre-DM reach the TC of the metformin after 6 and 15 weeks of the therapy of 1500 mg/day regimen.

## Methods

### Participants, patient’s diagnosis and treatment regimen

Compliance with Ethics Guidelines- The study was performed in accordance with the Helsinki Declaration of 1964, and its later amendments. Written informed consent was obtained from all the participants and the study was approved by the Medical University Bioethical Committee in Wroclaw, Poland (approval number: KB385/2017).

Treatment-naïve patients (regarding to hypoglycemic drugs), Caucasians, with new-diagnosed pre-diabetes based on fasting glucose and OGTT (oral glucose test tolerance) results were qualified for treatment (by main researcher-diabetologist) with immediate-release (regular form) metformin for 15 weeks (the period included 3 weeks titration) with final dose 1500 mg/day (3 × 500 mg). During titration period and blood collection visits, patients were asked about metformin tolerance and compliance. The patients were also encouraged to contact (telephone or personal) in any case of the problem with metformin tolerance.

The diagnosis of pre-DM was constituted if: FG was between 100–125 mg/dl ( 5.6- 6.9 mmol/L) for IFG (impaired fasting glycaemia) and/or after 2 h of 75 glucose solution taken the glucose was between 140–199 mg/dl (7.8–11.0 mmol/L) for IGT (impaired glucose tolerance), according to the local as well as European Association for the Study of Diabetes criteria^17^.

If potential side effects appeared, the patients could take a lower, well-tolerated dose of the drug.

Patients younger than 40 are rather encourage only to behavioral treatment in everyday practice so the authors did not decide to include them into the study. The risk for any cardio-vascular diseases in elderly population, defined as people over 65 yo^18^, is higher than in the younger population. Since the risk of heart failure increases with age and is a contraindication for the metformin use at any stage, the authors decided on this upper age limit (similar to the United Kingdom Prospective Diabetes Study).

Finally, inclusion criteria were: patients age: 40–65 years, with newly diagnosed pre-diabetic status based on fasting glucose and / or OGTT, without metformin or other hypoglycemic treatment before, without ischemic heart disease and/or stroke and/or PAOD (peripheral arterial occlusive disease) in a history.

Exclusion criteria for the patients were: diabetes, taking metformin or other anti-diabetics ever before the study, active cancer as could have the potential impact on metformin dosing and tolerance, history of macro-angiopathy (ischemic heart disease, stroke or transient ischemic attack, PAOD); serious gastrointestinal disease that may affect the metformin tolerance, important renal failure (eGFR, estimated glomerular filtration ratio, lower than 30 ml/min based on creatinine result), AlaT (alanin transaminase) > 3 × upper normal limit. The biochemical exclusion criteria (eGFR and AlaT) were based on European Medicines Agency^19^ and local guidelines^20^ for the metformin use.

The patients were recruited from Diabetes Centre Nowy Dwor between October 2017 and December 2018.

### Blood sampling and data for baseline characteristics

The blood samples from the patients were taken after 6 and 15 weeks (± 2 days) of the treatment to assess the random metformin concentration. Every time, beyond 3 weeks of the dose titration, next 3 weeks of target dose was established before the metformin concentration was assessed. The time, passed from the last dose of metformin taking, for patients who were visiting laboratory for blood taking, was not define and precisely recorded, but patients visited the Centre between 08.00 a.m. and 02 p.m., after the morning dose of the metformin was taken with breakfast, thus the time passed from the metformin intake was around 1–6 h^21,22^.

Before taking the first dose of the drug also information about baseline parameters like: lipids profile, AlaT, creatinine, HbA1c if available, was collected from the patients’ records, as well as weight, height, BMI and information about fatty liver based on the available ultrasonography organs’ description.

### Serum concentration of the metformin assessment and therapeutic range definition

Determination of metformin serum concentration was done by LC/MS (liquid chromatography-mass spectrometry) technique.

#### Sample preparation

Blood serum was obtained by centrifugation at 1040 RCF (relative centrifugation force) for 15 min at 4 °C in blood collecting tubes with calcium as precipitation enhancer.

100 µl 10% NaOH solution, 10 µl of internal standard (IS) solution (metformin-d6, 124 µM in water), 400 µl of acetonitrile and finally 10 µl of benzoil chloride solution (10% (v/v) in acetonitrile) were added to 100 µl of blood serum. The mixture was incubated for 10 min at 35 °C. Then mixture was centrifuged at 17,000 RCF at 4 °C.

100 µl of supernatant was moved to the chromatographic vial and 400 µl of water was added to create a sample.

#### LC/MS analysis

Measuring was performed using Agilent LCMS 6470 Triple Quadrupole tandem mass spectrometer equipped with Agilent UHPLC (Ultra-High-Pressure Liquid Chromatography), model Infinity 1290.

Injection volume of 1 µl of the aforementioned sample was taken, sample temperature in autosampler was 10 °C. The Waters BEH Shield C18 column (1.7 µm, 2.1 × 10 mm) was thermostated in column oven at 40 °C. Used flowrate was 0.5 ml/min. Used eluents were: A: water with 0.1% formic acid (FA), B: methanol with 0.1% FA. The gradient was as follows: 0.0 min—10% B, 0.5 min—10% B, 2.0 min—90% B, 3.5 min—90% B, 3.51 min—10% B, 7.0 min—10% B.

Electrospray ionization (ESI) was performed in positive ionization mode at 4 kV voltage while nozzle voltage was set to 0 kV with source gas 6 L/min at 250 °C and sheath gas 11 L/min at 350 °C.

Acquisition timeframe was 1.4–2.8 min. The multiple reaction monitoring (MRM) was performed. For analyte (metformin) reactions of precursor ion 216.1 Da to → 71.1, → 104.0, → 96.1 and → 56.2 Da fragments were observed while for IS (metformin-d6) reactions of precursor ion 222.1 Da to → 77.1, → 104.0, → 102.1 and → 59.2 Da fragments. The MRMs 216.1 → 71.1 Da for analyte and 222.1 → 77.1 Da for IS were used for quantification. Five-point calibration curve was performed (0.5, 2.0, 5.0, 10.0, and 20.0 µM; R2: 0.994)^23^.

#### The therapeutic range for the metformin

The therapeutic range for the metformin serum concentration was extrapolated from the studies on diabetes which were related to glycemia. Although the therapeutic serum concentration for metformin is not well defined^14^ and come on limited data, based on few studies it could be find as range from 0.1 to 20.7 (median 4.5) μmol/L^13,15–17^, if the kidney function is not disturbed, for the median drug’s dose 1500 mg/day.

According to the DPP study we defined adherence to metformin as taking at least 80% of assigned study pills^24^.

### Additional analysis

As additional analysis, the correlation between BMI and patient’s weight with serum metformin concentration was also performed.

### Statistical analysis

The Statistica 13 program was used with cut-off point for statistical significance (P) at 0.05. In order to determine the significance, statistical tests were used in accordance with the distribution of variables and the nature of the data (Student t test, Mann–Whitney test, The Wilcoxon signed-rank test). To determine the correlation the Spearman's rank-order correlation and the Pearson correlation was used.

## Results

**Figure 1:**
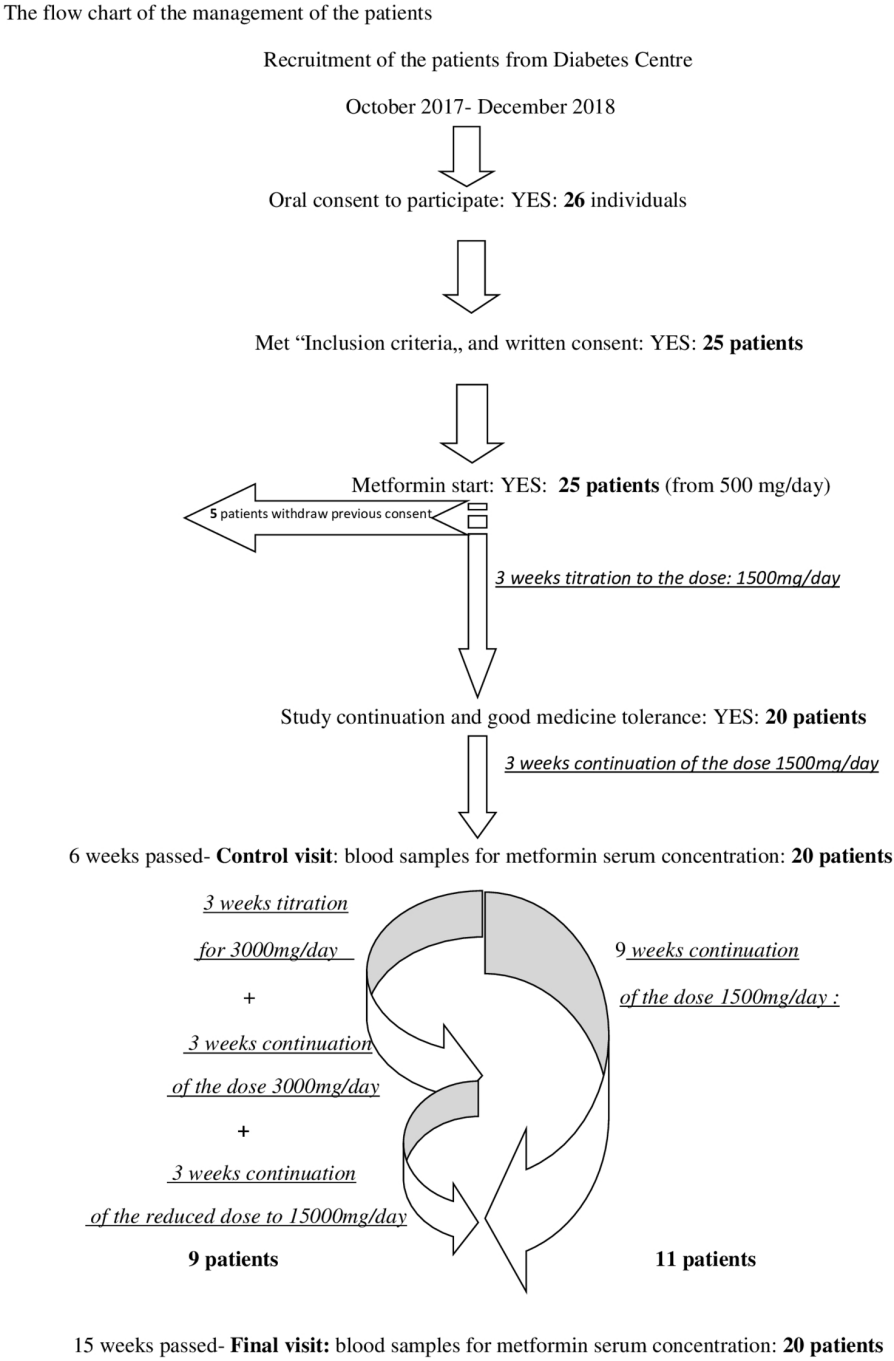
The flow chart of the management of the patients.

During the mentioned period, 26 people suspected for carbohydrate disturbances, by general practitioner, had given oral consent to participate in the study. After obtaining the blood results, diabetes diagnosis was constituted for one candidate, and the patient was disqualified from the further study procedure. Twenty five patients signed consent form to participate in the study, but 5 of them canceled their previous consent after few days because they found medication regimen (three times a day) or the study schedule too much absorbing in the context of their everyday duties. Finally 20 patients with new-diagnosed pre-DM were treated with the final metformin dosage 1500/day for 15 weeks, but 9 of them temporally (for 3 weeks) took higher dose: 3000 mg/day (3 × 1000 mg). However these 9 patients had wash-out period with dose reduction to 1500 mg/day, before the final metformin concentration was assessed. None of the patients reported significant side effects of the drug. Compliance to the metformin was 100% for the group, although several patients reported mild gastrointestinal complaints during the first week of the treatment. These symptoms resolved after diet modification within a few days. The flow chart of patients’ recruitment and description of the metformin treatment is attached as the Fig. 1.

The number of the males was 15 (75%), the mean patients’ age was 53.8 (40–64). Baseline parameters for studied group are shown in Table 1.

**Table 1:** Baseline parameters and lab-tests results for studied group.

Total number of patients	20
IFG_1_ number of patients/[%]	13/[65]
IGT_2_ number of patients/[%]	1/^5^
IFG + IGT number of patients/[%]	6/^30^
BMI_3_, (kg/m2) Mean/[SD_4_]/First quartile-Median-Third quartile	31.21/[4.68]/27.75–30.05–40.20
Weight (kg) Mean/[SD]/ First quartile-Median-Third quartile	94.80 /[12.45]/84.5–93.00–101.50
TCL_5_ (mg/dl) Mean/ [SD]/ range	196.55/ [48.24]/133–323
LDL_6_ (mg/dl) Mean/[SD]/range	117.42/[31.37]/81–207
HDL_7_ (mg/dl) Mean/[SD]/range	51.55/[13.39]/32–80
TG_8_ (mg/dl) Mean/[SD]/range	118.95/[45,32]/55–227
AlaT_9_ (U/l) Mean/[SD]/range	30.55/[12.79]/8–61
Creat_10_ (mg/dl) Mean/[SD]/range	0.86/[0.15]/ 0.59–1.2
eGFR_11_ (ml/min/m^2^) Mean/[SD]/range	94.42/[16.28]/67.17–124.57
HbA1c_12_ (%) / (mmol/mol) Mean/[SD]/range	5.7 / 39/[0.69]/4.8–7.5% /29–58
Fatty liver number of patients/[%]	12/[60]

_1_Impaired fasting glucose; _2_impaired glucose tolerance; _3_body mass index; _4_standard deviation, _5_total cholesterol level, _6_low density lipoprotein; _7_high density protein; _8_triglycerides; _9_alanine transaminase; _10_creatinine; _11_estimated glomerular fraction; _12_glycated hemoglobin.

All the patients had fasting plasma glucose tests, OGTT, lipid parameters, AlaT, creatinine and ultrasonography of the abdomen performed, but HbA1c was available only for 13 participants, as it is not a routine test in diabetes diagnosis according to the local guidelines^20^. The baseline results of the cholesterol level, kidney function, HbA1c and liver parameters are shown in Table 1. One patient reach HbA1c > 6.4% (46 mmol/mol), what is considered as a value for diabetes, but presented results typical for glucose intolerance and not for diabetes.

**Figure 2:**
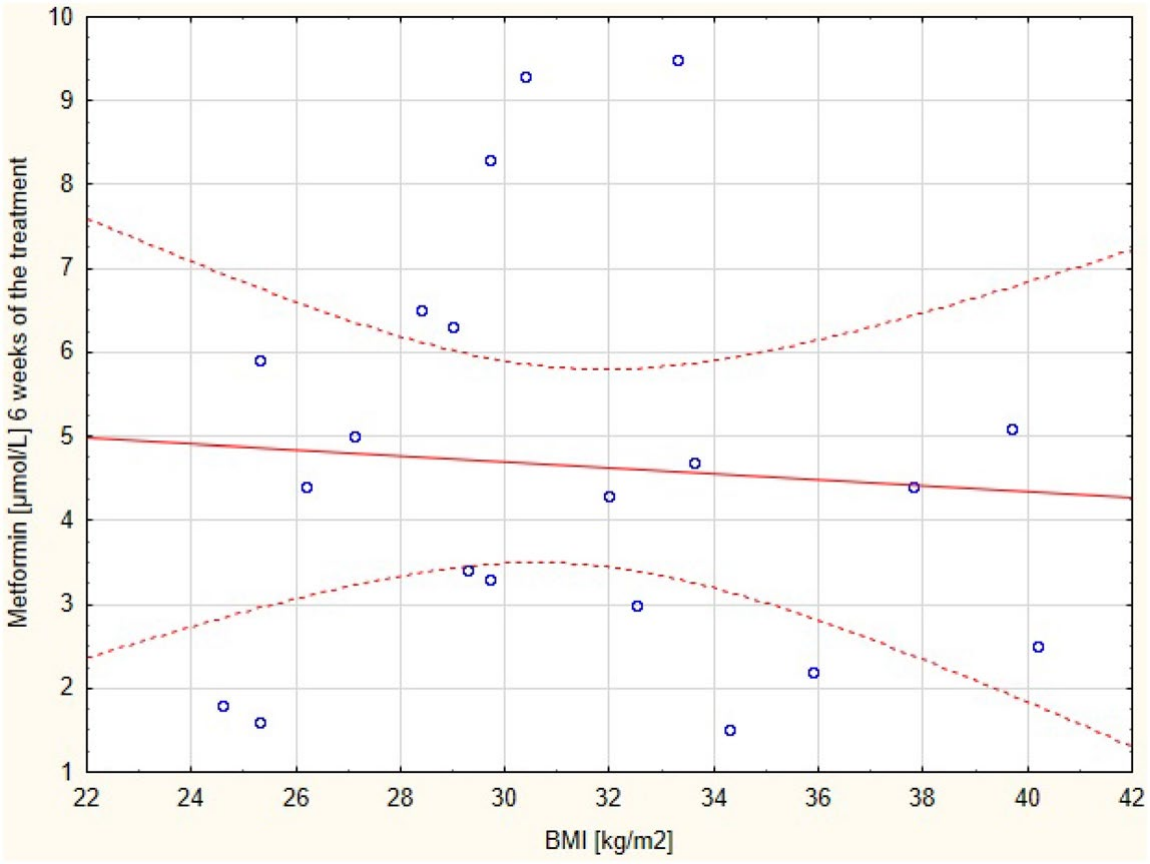
Correlation between metformin concentration after 6 weeks of the treatment with BMI.

The Total ion current (TIC) and The multiple reaction monitoring (MRM) for metformin-d6 and metformin are in the Supplementary Materials (Figs. 1 and 2 respectively). The Calibration curve for the LC–MS/MS analysis of metformin is in Supplementary materials Fig. 3.

**Figure 3:**
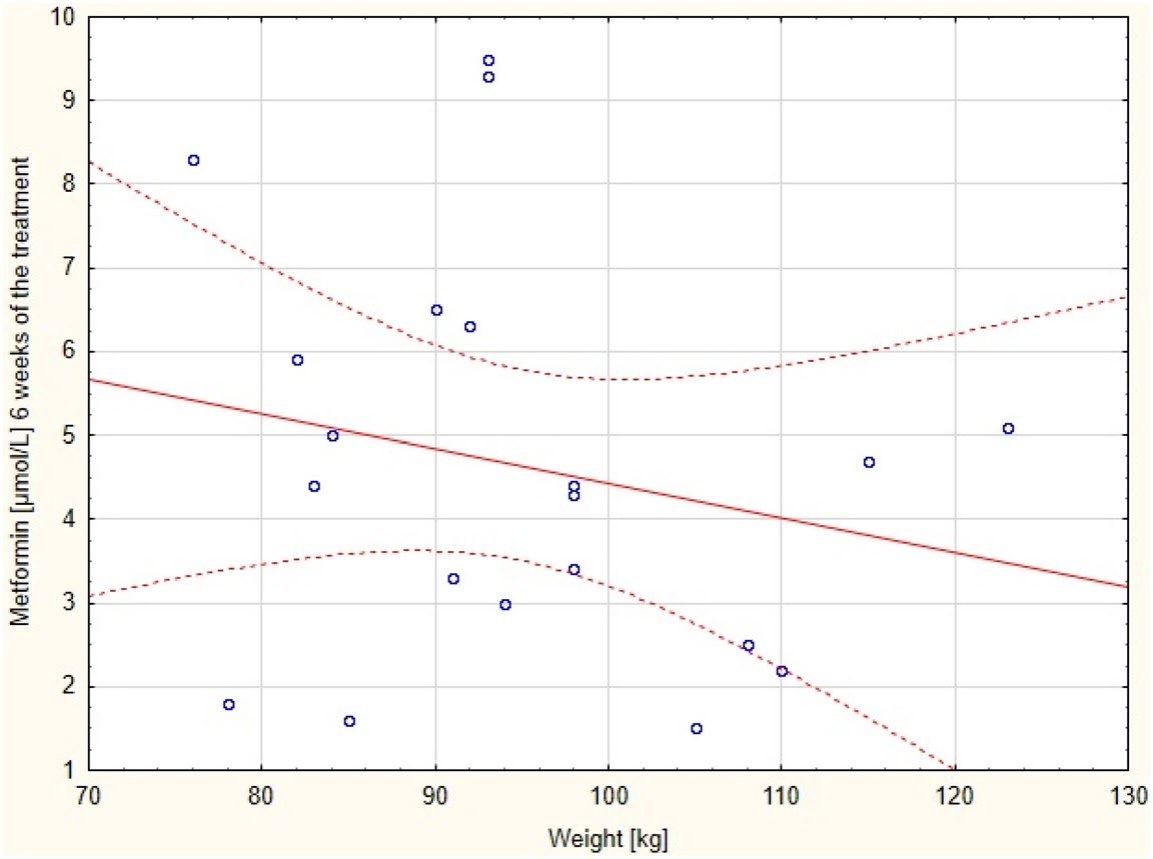
Correlation between metformin concentration after 6 weeks of the treatment with body weight.

Metformin serum concentration after 6 and 15 weeks of the treatment was similar within the group and all the patients reached the proposed therapeutic concentration after only 6 weeks of the therapy (Table 2).

**Table 2:** Metformin serum concentration after 6 and 15 weeks of the treatment.

Descriptive statistics									
Prameter	Number of patients	Mean	SD_1_	First quartile	Median	Third quartile	Min	Max	p
Metformin [μmol/L] 6 weeks of the treatment	20	4.65	2.41	2.75	4.40	6.10	1.50	9.5	0.27
Metformin [μmol/L] 15 weeks of the treatment	20	5.41	3.44	3.30	4.75	6.75	1.20	15.10	

SD_1_- standard deviation, p- statistical significance.

**Figure 4:**
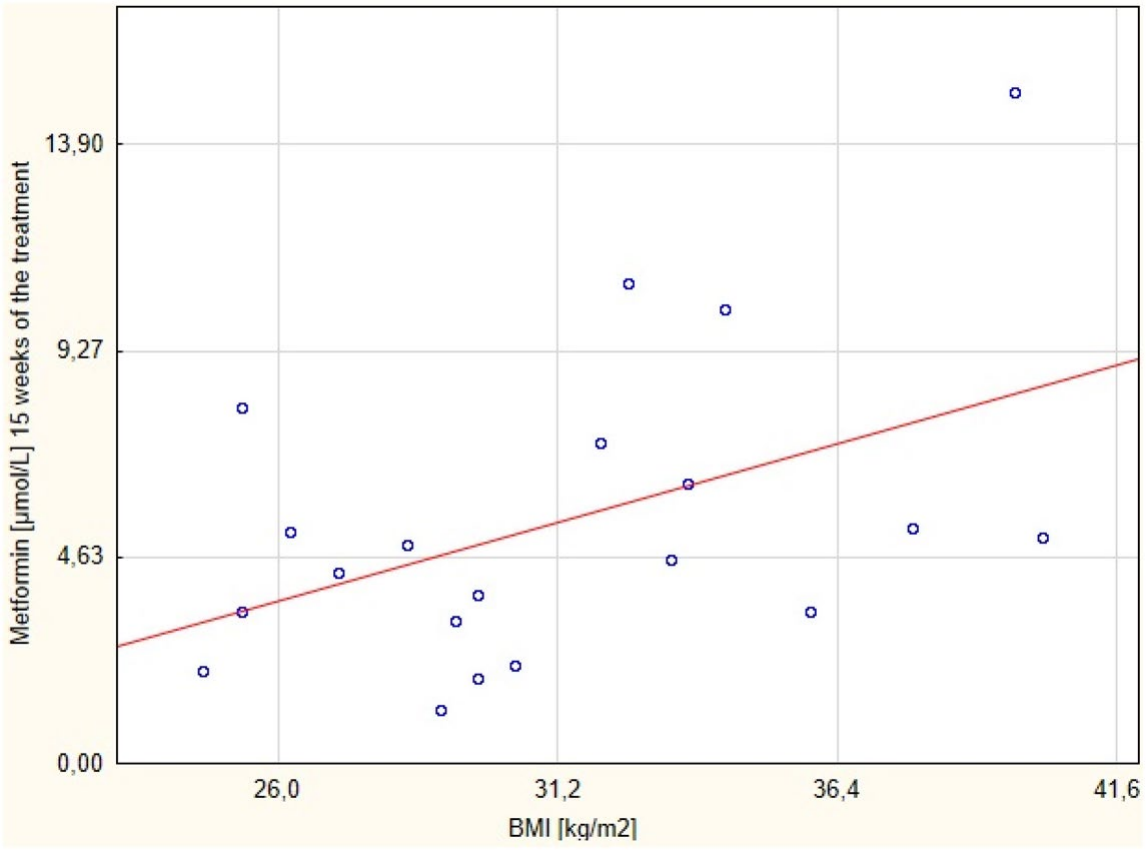
Correlation between metformin concentration after 15 weeks of the treatment with BMI.

**Figure 5:**
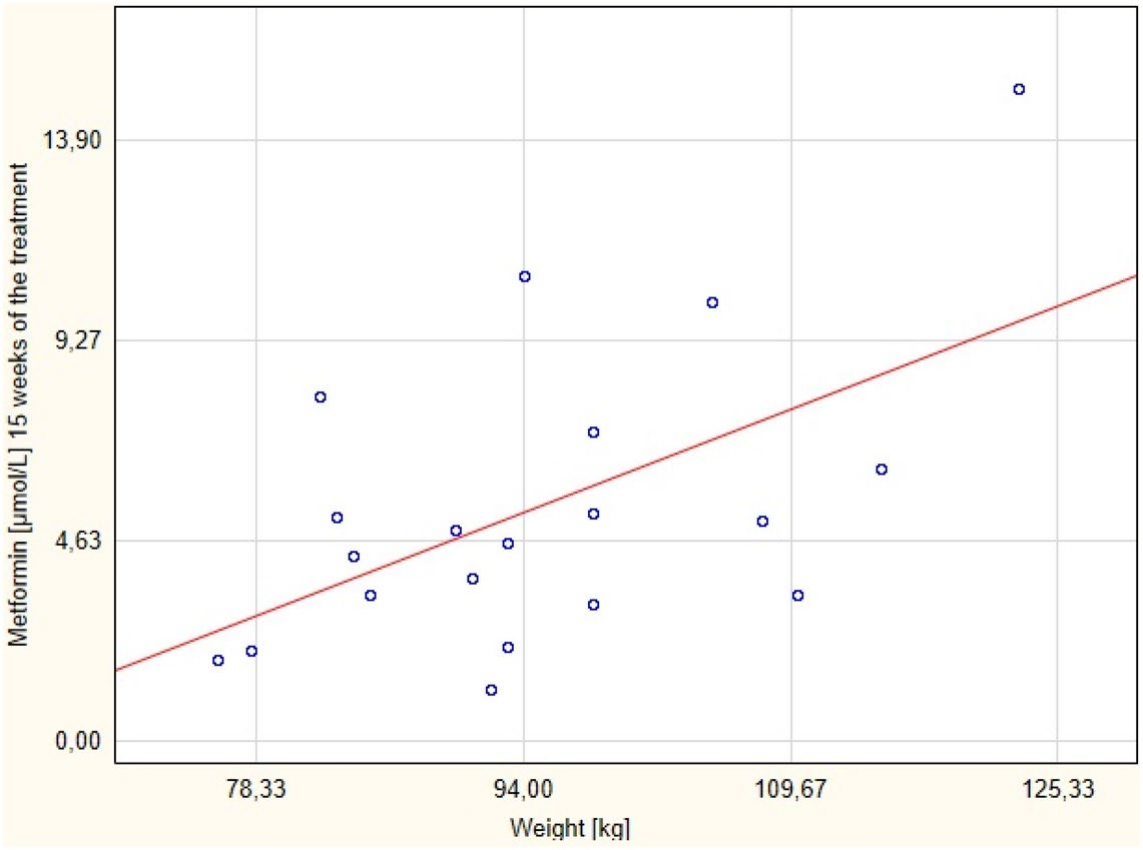
Correlation between metformin concentration after 15 weeks of the treatment with body weight.

The correlations of the serum drug concentration with BMI and body weight, during the proposed metformin regimen after 6 (Figs. 2 and 3 respectively) and 15 (Figs. 4 and 5 respectively) weeks of the treatment were assessed.

There was positive correlation between the serum concentration of the metformin (but not BMI) and the body weight after 15 weeks of the therapy. No correlation between these parameters was found after 6 weeks of the treatment. The correlation between these variables after 6 and 15 weeks are presented in Tables 3 and 4, respectively.

**Table 3:** Correlation between metformin concentration after 6 weeks of the treatment with BMI and body mass.

Parameter	Correlation				
	Mean	SD	r(XY)	p	Number of the parameters
Metformin [μmol/L] 6 weeks of the treatment	4.65	2.41	−0.069	0.77	20
BMI [kg/m^2^]	31.21	4.68			
Metformin [μmol/L] 6 weeks of the treatment	4.65	2.41	−0.213	0.36	20
Weight [kg]	94.80	12.45			

SD- standard deviation; r(XY)- Pearson correlation; p- statistical significance.

**Table 4:** Correlation between metformin serum concentration after 15 weeks of the treatment with BMI and body mass.

Pair of variables	Spearman's rank-order correlation		
	Number of the parameters	R Spearman	p
Metformin [μmol/L ] 15 weeks of the treatment & BMI [kg/m^2^]	20	0.42	0.06
Metformin [μmol/L ] 15 weeks of the treatment & weight [kg]	20	0.45	0.04

BMI- body mass index; R Spearman- Spearman's rank-order correlation; p- statistical significance.

## Discussion

During the study the adherence to the drug was excellent what gives the researchers opportunity to assess the impact of the low dose of the metformin on the results. The study has shown that after quite short period of the treatment (6 weeks after treatment was started, 4 weeks of the final regimen 1500 mg/day) patients with pre-diabetes reached the therapeutic metformin concentration, which is attributed to the glycemic effect in diabetic population and based on previous studies^14^. In DPP study, where the adherence to metformin was 72% ^1^ , the participants took the metformin with a total daily dose 1700 mg/day, which was shown to give the patients glycemic benefit. Whether the daily dose, used in our study (1500 mg/day), protect patients from diabetes is unclear and it should be studied in a bigger group of the patients with pre-DM with long-term drug use, however the metformin serum concentration reached in the study therapeutic concentration according to the biochemical suggestions^14^.

The blood metformin concentration at steady state depends on: the dose, bioavailability, total drug clearance, volume of distribution and interval between doses^25^, and shows relatively low variability in individual but high interindividual variability^26^. It was also find in our study. The difference of the metformin serum concentration varied around 6–12-fold (see minimum and maximum concentration in Table 2). Some of our patients took statins and Angiotensin-converting-enzyme inhibitors or Angiotensin II receptor blockers but these medicines are not involved in interactions with studied drug and thus could not impact the metformin concentration results^15^. The metformin in our study was taken as 1500 mg/day of the regular form (three times a day 500 mg with the time gaps declared by the participants were around 8–10 h). This suggests that bioavailability, total clearance and volume of the distribution may have had an impact on the metformin serum concentration. None of the patients in our study had GFR lower than 60 ml/min and, as was clarified at the exclusion criteria, we tried to exclude the potential gastrointestinal problems (clinically important liver disease or metformin intolerance) which could have an impact e.g. on substance absorption. Aware that we cannot influence and detect all the factors that modify the bioavailability of the drug (e.g. impact of the food) every day, we decided to find if body weight and /or BMI could affect the serum drug concentration. In previous study it was shown that body weight is a predictor for the volume distribution and thus can impact the serum concentration of the metformin^27,28^. The results after 15, but not 6, weeks of the treatment have shown us positive correlation between the metformin serum concentration and body weight (but not BMI) what can suggest that long-duration therapy can modify drug serum concentration. In our study the higher body weight was, the higher concentration of the metformin was observed. Interesting is that after 6 weeks of the treatment the Pearson correlation was negative, however, weak and without statistical significance. After next weeks of the therapy the correlation reversed and was positive with moderate strength (without statistical significance for BMI, but with statistical significance for body weight). The question is what happened. Because the group was small and because of the other, mentioned below, limitation of the study we can only speculate. Generally BMI should not affect hydrophilic drugs’ volume of distribution, which includes metformin, but in obese individuals increased eGFR and tubular secretion can be responsible for higher drug elimination^28,29^. This observation from previous studies can explain the results after 6 weeks of the treatment in our research. However, the impact of the treatment duration on metformin concentration has also been suggested. After a long period of using metformin, the important role of the deep compartment can be seen. One study confirmed deceleration of average metformin elimination after 6 weeks of the therapy due to distribution in the deep compartment (erythrocytes)^30^, so the relationship between drug concentration and baseline patient parameters may differ from those previously known if we consider the duration of the therapy. This may have important clinical implications in the context of the pleiotropic effect.

The problem with interpretation of this data is due to the fact that the last metformin dose administration time was not recorded, therefore potentially the trough, as well as the peak metformin concentration could be found. The random blood sampling resulted from patient’s availability declaration. This relatively young, professionally active individuals could not have been visiting laboratory at the time corresponding to minimum and/or maximum serum drug concentration, because of their time constraint. However, a six-week period is sufficient to establish steady-state drug concentration, and possible fluctuations should be within the recommended therapeutic range if a constant dose and similar time interval are maintained. Thus, the interpreted data reveal the metformin concentration at steady-state what reflects every day treatment effects, but the lack of blood sampling regimen can bias the potential impact of the body weight on the results. Because metformin alters insulin sensitivity in the human body, with all its consequences^12^, the possible impact of this (e. g. correlation with endogenous insulin or C-peptide concentration) on the observed correlation cannot be ruled out and should be followed in future studies.

Because of a small group and disproportion between males (75%) and females we did not assess the impact of the sex on the metformin serum concentration, although there are studies that confirmed sex differences in drug blood concentration^31–33^. The serum metformin concentration may be also influenced by some renal transporters. This was described in detail by Graham et al.^15^. Some of single nucleotide polymorphisms found in genes which encoding organic cation transporters and multidrug and toxin extrusion transporter are associated with observed lower serum metformin concentration in some subjects. However, the impact of cationic transporters on clinical response in patients treated with metformin is rather limited.

All the participants with pre-diabetes reached the therapeutic metformin serum concentration after only 6 weeks of the 1500 mg/day therapy and none of them reached the drug concentration above therapeutic limit, assigned to the patients with diabetes in previous studies. It means that even at such a low dose of drug, patients can be successfully treated in mind of the biochemical parameters, what was never shown before (in DPP study the dose was 1700 mg/day). To define if the obtained concentration is beneficial to the individuals with pre-DM, gives the clinical effect and thus could be constituted as a therapeutic concentration for this group of patients, plasma glucose and other parameters describing patients' metabolic status should be assessed as effectiveness biomarkers. The impact of body weight on metformin blood concentration cannot be excluded after longer duration of the treatment. The aforementioned effectiveness of the low-dose therapy as well as the impact of the treatment duration on the correlation of various patient parameters with drug concentration requires long-term studies on a large number of patients. These could be clinically important in the context of pleiotropic effect of the metformin e.g. it is more difficult to answer the question what we consider as the therapeutic dose for a drug with pleiotropic effect e.g. antioxidant activity, on cardio-vascular complications.

### Limitation of the study

The small number of the participants is the main limitation of the study. The assessment of a random metformin concentration may also limit the analysis of correlation with parameters.

## Conclusions

Patients with pre-diabetes can be successfully treated with a low dose of metformin, to reach drug’s therapeutic concentration. Body weight can impact the metformin serum concentration during long-term treatment what should be taken into consideration when choosing the dose because of its pleiotropic effect e.g. on cardiovascular system via reduction the oxidative stress, and would be not connected with the drug’s hypoglycemic effect.

## Supplementary Information

Supplementary Information 1.

Supplementary Information 2.

Supplementary Information 3.

## Data Availability

The data will be available at: https://pl.padlet.com/edytasutkowska/pddamutypcfn53do. The data were presented at the 5th Cardiovascular Outcome Trial (CVOT) Summit. Munich (Germany), 24th-25th October 2019 as a poster.

## References

[1] description of lifestyle intervention (2002). Diabetes Prevention Program (DPP) Research Group. The Diabetes Prevention Program (DPP). Diabetes Care.

[2] the Diabetes Prevention Program Outcomes Study (2015). Diabetes Prevention Program (DPP) Research Group. Long-term effects of lifestyle intervention or metformin on diabetes development and microvascular complications over 15-year follow-up. Lancet Diabetes Endocrinol..

[3] Diabetes Prevention Program (DPP) Research Group. Long-term Effects of Metformin on Diabetes Prevention: Identification of Subgroups That Benefited Most in the Diabetes Prevention Program and Diabetes Prevention Program Outcomes Study. *Diabetes Care* **42**, 601–608. 10.2337/dc18-1970 (2019). 10.2337/dc18-1970 PMC6429636 30877090

[4] an intent-to-treat analysis of the DPP/DPPOS (2012). Diabetes Prevention Program (DPP) Research Group. The 10-year cost-effectiveness of lifestyle intervention or metformin for diabetes prevention. Diabetes Care.

[5] Goldberg R (2013). Lifestyle and metformin treatment favorably influence lipoprotein subfraction distribution in the Diabetes Prevention Program. J. Clin. Endocrinol. Metab..

[6] Goldberg R (2014). Diabetes Prevention Program Research Group. Lifestyle and metformin interventions have a durable effect to lower CRP and tPA levels in the Diabetes Prevention Program except in those who develop diabetes. Diabetes Care.

[7] the Diabetes Prevention Program randomized trial (2005). Orchard, T. *et al*. Diabetes Prevention Program (DPP) Research Group. The effect of metformin and intensive lifestyle intervention on the metabolic syndrome. Ann. Intern. Med..

[8] Goldberg R (2017). Diabetes Prevention Program (DPP) Research Group. Effect of long-term metformin and lifestyle in the Diabetes Prevention Program and its outcome study on coronary artery calcium. Circulation.

[9] UK Prospective Diabetes Study (UKPDS) Group. Effect of intensive blood-glucose control with metformin on complications in overweight patients with type 2 diabetes (UKPDS 34). *Lancet.* **352**, 854–865 (1998). 9742977

[10] Hong J (2013). SPREAD-DIMCAD Investigators. Effects of metformin versus glipizide on cardiovascular outcomes in patients with type 2 diabetes and coronary artery disease. Diabetes Care.

[11] Kooy A (2009). Long-term effects of metformin on metabolism and microvascular and macrovascular disease in patients with type 2 diabetes mellitus. Arch. Intern. Med..

[12] Metformin 500 mg. Available from: https://www.medicines.org.uk/emc/product/594/smpc. Accessed 17 Feb 2020.

[13] Frid A (2010). Novel assay of metformin levels in patients with type 2 diabetes and varying levels of renal function. Diabetes Care.

[14] Kajbaf F, De Broe M, Lalau J (2016). Therapeutic concentrations of metformin: a systematic review. Clin. Pharmacokinet..

[15] Graham G (2011). Clinical Pharmacokinetics of metformin. Clin. Pharmacokinet..

[16] Lalau J (1995). Role of metformin accumulation in metformin-associated lactic acidosis. Diabetes Care.

[17] Lalau J, Lacroix C (2003). Measurement of metformin concentration in erythrocytes: clinical implications. Diabetes Obes Metab..

[18] The Task Force for diabetes, pre-diabetes, and cardiovascular diseases of the European Society of Cardiology (ESC) and the European Association for the Study of Diabetes (EASD). 2019 ESC Guidelines on diabetes, pre-diabetes, and cardiovascular diseases developed in collaboration with the EASD. *Eur. Heart. J.* **41**, 255–323. 10.1093/eurheartj/ehz486 (2020). 10.1093/eurheartj/ehz486 31497854

[19] World Health Organization. Definition of an older or elderly person. http://www.who.int/healthinfo/survey/ageingdefnolder/en/index.html. Accessed 17 Feb 2020.

[20] Metformin and metformin-containing medicines. EMA. https://www.ema.europa.eu/en/medicines/human/referrals/metformin-metformin-containing-medicines. Accessed 17 Feb 2020.

[21] 2017 Guidelines on the management of diabetic patients. A position of Diabetes Poland. *Clin. Diabetol.* **6**(Suppl. A), 1 (2017).

[22] Azar J, Bassil M, Irani J, Badawi K, Sawan C (2018). Factors determining blood and urine concentrations of metformin among patients with type 2 diabetes: A Cross Sectional Study. J Biomed. Sci.

[23] Wiśniewski J (2017). A novel mass spectrometry-based method for simultaneous determination of asymmetric and symmetric dimethylarginine, l-arginine and l-citrulline optimized for LC-MS-TOF and LC-MS/MS. Biomed. Chromatogr..

[24] Knowler W (2002). Diabetes Prevention Program Research Group. Diabetes Prevention Program Research Group. Reduction in the incidence of type 2 diabetes with lifestyle intervention or metformin. N. Engl. J. Med..

[25] Stage T (2015). A twin study of the trough plasma steady-state concentration of metformin. Pharmacogenet. Genom..

[26] Christensen M (2011). The pharmacogenetics of metformin and its impact on plasma metformin steady-state levels and glycosylated hemoglobin A1c. Pharmacogenet. Genomics..

[27] Duong J (2013). Population pharmacokinetics of metformin in healthy subjects and patients with type 2 diabetes mellitus: simulation of doses according to renal function. Clin. Pharmacokinet..

[28] Bardin C (2012). Population pharmacokinetics of metformin in obese and non-obese patients with type 2 diabetes mellitus. Eur. J. Clin. Pharmacol..

[29] Ghobadi C (2011). Application of a system approach to the bottom-up assessment of pharmacokinetics in obese patients: expected variations in clearance. Clin. Pharmacokinet..

[30] Ningrum V, Ikawati Z, Sadewa A, Ikhsan M (2018). Patient-factors associated with metformin steady-state levels in type 2 diabetes mellitus with therapeutic dosage. J. Clin. Transl. Endocrinol..

[31] Chen M (2000). Pharmacokinetic analysis of bioequivalence trials: implications for sex-related issues in clinical pharmacology and biopharmaceutics. Clin. Pharmacol. Ther..

[32] Freire A, Basit A, Choudhary R, Piong C, Merchant H (2011). Does sex matter? The influence of gender on gastrointestinal physiology and drug delivery. Int. J. Pham..

[33] Soldin O, Mattison D (2009). Sex differences in pharmacokinetics and pharmacodynamics. Clin. Pharmacokinet..

