# Supplementary figures and images for: Low metformin dose and its therapeutic serum concentration in prediabetes
Source: Sci Rep. 2021 Jun 3;11:11684. doi: 10.1038/s41598-021-91174-7 (PMC8175603; doi:10.1038/s41598-021-91174-7)

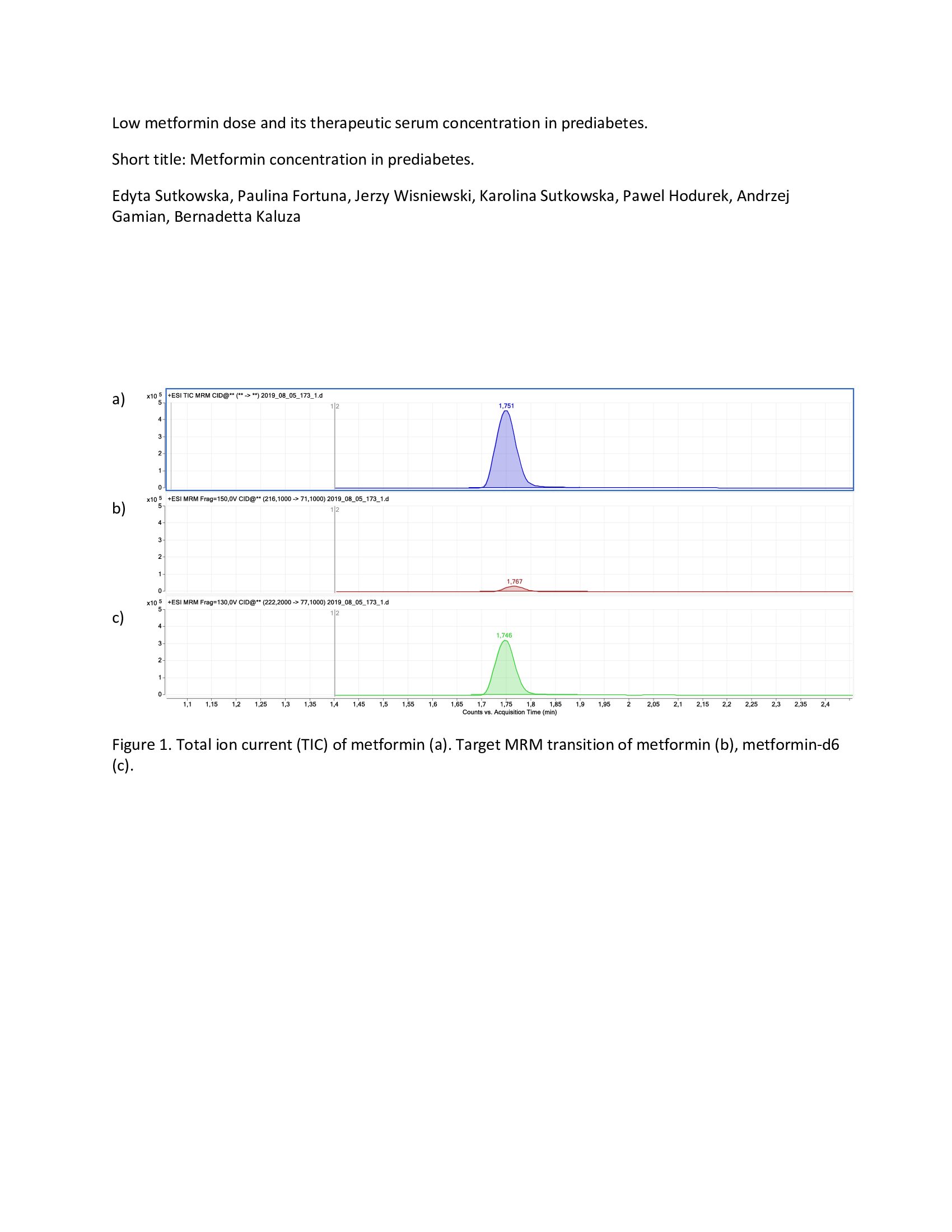

Supplement: Supplementary file 1 — Supplementary Information 1. [file 41598_2021_91174_MOESM1_ESM.jpg]

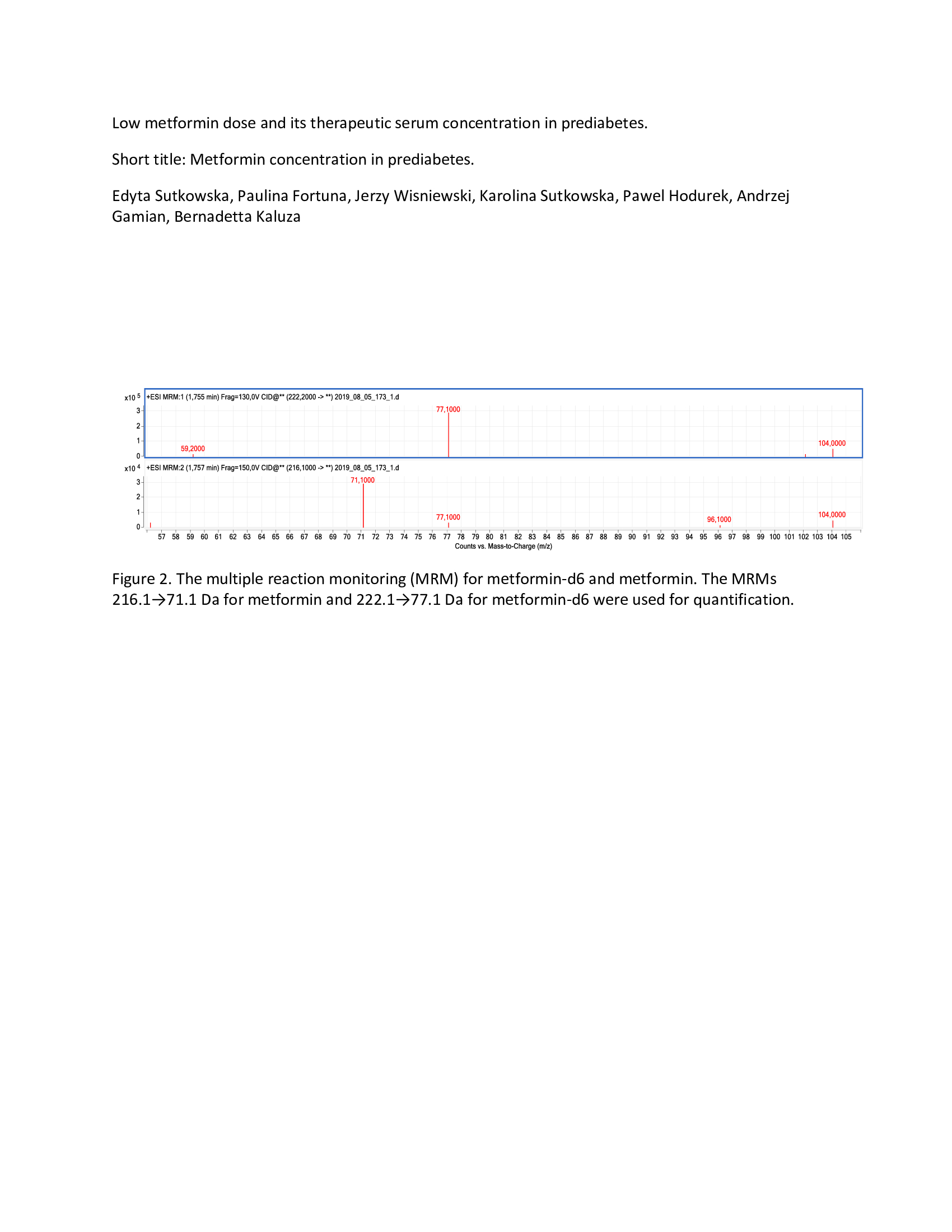

Supplement: Supplementary file 2 — Supplementary Information 2. [file 41598_2021_91174_MOESM2_ESM.jpg]

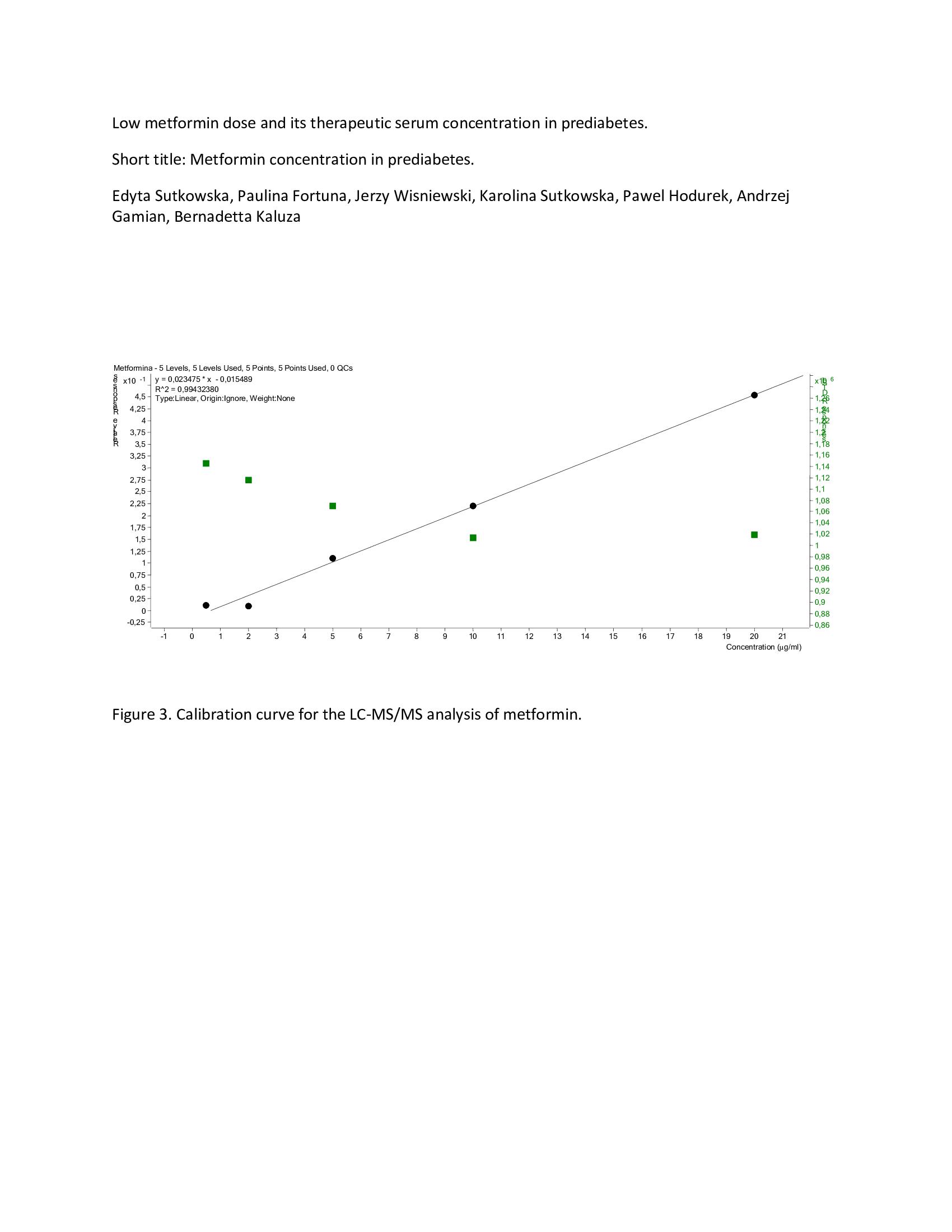

Supplement: Supplementary file 3 — Supplementary Information 3. [file 41598_2021_91174_MOESM3_ESM.jpg]
